# The Association between Vascular Abnormalities and Glaucoma—What Comes First?

**DOI:** 10.3390/ijms241713211

**Published:** 2023-08-25

**Authors:** Xiaosha Wang, Maoren Wang, Hanhan Liu, Karl Mercieca, Julia Prinz, Yuan Feng, Verena Prokosch

**Affiliations:** 1 Department of Ophthalmology, University of Cologne, Kerpener Str. 62, 50937 Cologne, Germany; xwang21@smail.uni-koeln.de (X.W.); wmaoren@163.com (M.W.); hanhan.liu@uk-koeln.de (H.L.); juprinz@ukaachen.de (J.P.); feng.yuan@uk-koeln.de (Y.F.); 2 Department of Ophthalmology, University Medical Center, Johannes Gutenberg University Mainz, Langenbeckstr. 1, 55131 Mainz, Germany; 3 Department of Ophthalmology, Beijing Chaoyang Hospital, Capital Medical University, Beijing 100020, China; 4 Glaucoma Section, University Hospital Eye Clinic, 53127 Bonn, Germany; karl.mercieca@ukbonn.de; 5 Faculty of Biology, Medicine and Health, School of Health Sciences, University of Manchester, Manchester M13 9WH, UK; 6 Department of Ophthalmology, RWTH Aachen University, 52074 Aachen, Germany

**Keywords:** glaucoma, vascular abnormalities, vascular biomarkers, neurodegeneration, intraocular pressure

## Abstract

Glaucoma is a leading cause of irreversible blindness worldwide. While intraocular pressure (IOP) presents a major risk factor, the underlying pathophysiology still remains largely unclear. The correlation between vascular abnormalities and glaucoma has been deliberated for decades. Evidence for a role played by vascular factors in the pathogenesis of glaucomatous neurodegeneration has already been postulated. In addition, the fact that glaucoma causes both structural and functional changes to retinal blood vessels has been described. This review aims to investigate the published evidence concerning the relationship between vascular abnormalities and glaucoma, and to provide an overview of the “chicken or egg” dilemma in glaucoma. In this study, several biomarkers of glaucoma progression from a vascular perspective, including endothelin-1 (ET-1), nitric oxide, vascular endothelial growth factor (VEGF), and matrix metalloproteinases (MMPs), were identified and subsequently assessed for their potential as pharmacological intervention targets.

## 1. Introduction

Glaucoma is characterized by the progressive death of retinal ganglion cells (RGCs) and consequential functional impairments of the visual field [1]. However, despite extensive research efforts, the precise pathogenesis of glaucoma remains inadequately understood. While elevated IOP has traditionally been recognized as the primary risk factor in glaucoma development, it is crucial to acknowledge that a significant proportion of patients experience disease progression despite successful normalization of IOP through therapeutic interventions [2].

There is a growing body of compelling evidence supporting the notion that vascular abnormalities, encompassing both vascular factors and systemic hemodynamics, play a substantial role in the complex etiology of glaucoma. The vascular theory in glaucomatous neurodegeneration postulates that diminished perfusion pressure, impaired vascular autoregulation, and disrupted neurovascular coupling serve as the underlying mechanisms. These factors collectively contribute to the progressive degeneration of the optic nerve and retinal ganglion cells in glaucoma [3,4].

Regarding hemodynamics, a notable association between glaucoma and systemic cardiovascular diseases, such as diabetes mellitus (DM) [5,6,7], high blood pressure (BP) [8,9], low BP [10], and nocturnal dipping [11] has been documented, suggesting an elevated incidence of glaucoma in affected individuals. These observations highlight the potential influence of hemodynamic factors on glaucoma development and progression [12,13]. Abnormal vascular function can also influence the secretion and drainage of aqueous humor (AH), thus affecting IOP regulation [14]. The elevated IOP resulting from vascular abnormalities can induce compression of RGCs and their axons, as well as diminished ocular blood supply. Consequently, these factors contribute to the pathogenesis and progression of glaucoma [15].

Retinal vascular dysfunction has been reported secondary to glaucoma, indicative of a bidirectional relationship between glaucoma and retinal vascular pathology [16]. These secondary manifestations of glaucoma include a reduction in retinal vascular diameter [17], decreased retinal vascular density [18], alterations in retinal vascular molecules [19], impaired autoregulation, arteriolar dysfunction within the retina [16], and even occurrences of retinal vein occlusion [17,20]. However, the answer to the question of whether vascular abnormalities drive glaucoma progression or if glaucoma serves as the instigator of vascular abnormalities remains elusive. In this comprehensive review, we investigate published evidence and provide a succinct summary of the vascular factors that potentially contribute to the pathogenesis of glaucoma. Additionally, we explore ocular vascular abnormalities that arise as a consequence of glaucoma. Furthermore, our analysis aims to identify potential vascular biomarkers that can serve as indicators for the progression and prognosis of glaucoma and the role of these biomarkers in refining current management strategies for glaucoma.

## 2. The Anatomy of Blood Supply in the Retina

**Figure 1:**
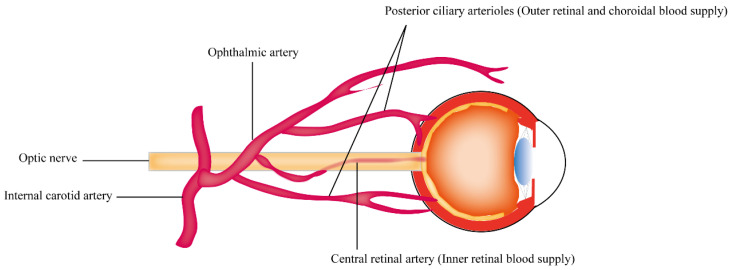
Anatomical depiction of the retinal blood supply.

The retina is a highly metabolically active tissue that requires a constant supply of nutrients and oxygen to function properly. The blood supply of the outer retina and inner retina comes from different circulatory pathways: the central retinal artery (CRA) and the choroidal circulation, respectively. This is summarized in Figure 1. The CRA is a branch of the ophthalmic artery, which arises from the internal carotid artery. The artery enters the eye through the optic nerve and travels through the center of the optic nerve to reach the retina. The CRA then divides into multiple smaller branches that supply blood to the inner retina, which includes the ganglion cells, inner plexiform layer, and inner nuclear layer [17,21]. The choroidal circulation is a network of blood vessels that lies between the retina and the sclera, the outermost layer of the eye. Blood from the choroidal circulation enters the retina through the outer retinal vessels and then flows towards the inner retina. The choroidal circulation supplies the outer retina, including the photoreceptors and the retinal pigment epithelium [22]. The high oxygen demands of the retina and the relatively sparse nature of the retinal vasculature are believed to be responsible for the particular vulnerability of the retina to vascular disease [22,23]. This indicates that neurons in the inner retina, such as RGCs, are susceptible to limited retinal inner blood supply in retinal diseases such as glaucoma and diabetic retinopathy [22,24]. Animal studies have demonstrated that bilateral occlusion of the common carotid arteries, leading to retinal hypoperfusion, causes a significant reduction in RGC density in rats due to apoptosis [25,26]. Clinical studies have also revealed a strong correlation between impaired ocular blood flow and glaucoma severity, particularly in patients with reduced perfusion in the optic nerve head (ONH) [27,28,29]. Such findings suggest that RGCs are particularly susceptible to reduced ocular blood flow.

## 3. The Role of Vascular Abnormalities in Glaucomatous Neurodegeneration

The vascular theory of glaucomatous neurodegeneration suggests that reduced perfusion pressure, faulty vascular autoregulation, and loss of neurovascular coupling play important roles in the relationship between vascular abnormalities and glaucomatous neurodegeneration [30,31]. In terms of hemodynamics, an increased incidence of glaucoma has been observed in patients with systemic cardiovascular diseases, such as DM [5,6] and high BP [8,9]. This observation underscores the potential impact of hemodynamic factors on the onset and advancement of glaucoma. Abnormal vascular function can also influence the secretion and drainage of aqueous humor, consequently increasing IOP and contributing to the pathogenesis and progression of glaucoma [14]. In this section, we provide a detailed discussion of these factors, elucidating their significance in the context of glaucoma. Additionally, we offer a comprehensive summary of the biomarkers that have been associated with glaucoma.

### 3.1. Ocular Perfusion Pressure and Its Role in Glaucomatous Neurodegeneration

The ocular perfusion pressure (OPP) refers to the pressure necessary to propel blood through the intraocular vasculature and represents the blood flow and oxygen supplying ONH [32]. Thus, it has long been proposed that a decrease in OPP may increase the vulnerability of the optic disc, leading to an increased risk of glaucoma development or progression [33,34,35,36]. In the Barbados Eye Study, individuals with low mean OPP exhibited a risk ratio of 2.6 for incident open-angle glaucoma (OAG) over a 9-year follow-up period [37]. The Singapore Malay Eye Study and the Los Angeles Latino Eye Study revealed a direct relationship between decreased OPP and increased prevalence of OAG [36,38]. The actual OPP should be determined by the difference between arterial pressure at the entrance to the eye and venous pressure at the exit of the eye. Since currently available methods cannot directly measure such pressures, OPP has been estimated by the difference between arterial pressure measured in the arm and IOP [33]. As a crucial modifiable part of OPP, systemic hemodynamics are supposed to play a role in the pathology and treatment of glaucoma, and this is further discussed in Section 3.4.

### 3.2. Vascular Autoregulation and Its Role in Glaucomatous Neurodegeneration

Vascular autoregulation, commonly known as vasoreactivity, represents the intrinsic capacity of the vascular system to adapt to varying physiological conditions and meet diverse metabolic demands by maintaining stable and sufficient blood flow [30,39]. A recent study suggested that the lower autoregulation limit is quite close to physiological OPP values [40]. This means that healthy optic nerves are prone to hypoperfusion with small fluctuations in BP or IOP. In pathological conditions [41], such as DM [42] and hypercholesterolemia [43], autoregulation in the ONH can be disrupted. In a study centered on autoregulation of the retrobulbar hemodynamics [44], it was observed that the unaltered flow velocities in the short posterior ciliary artery (SPCA) of healthy individuals, as a response to postural changes, suggest a strict autoregulatory control. However, in contrast, patients with normal-tension glaucoma (NTG) and primary open-angle glaucoma (POAG) demonstrated an insufficient compensatory response to postural changes. The authors of the study suggest that compromised autoregulatory control may represent another contributing factor in the pathogenesis of glaucomatous neurodegeneration. In fact, the disruption of vascular autoregulation in ONH has been reported to play a role in the etiology and progression of glaucoma for decades [30,45,46,47].

### 3.3. Neurovascular Coupling and Its Role in Glaucomatous Neurodegeneration

The central nervous system exhibits highly coordinated coupling between neuronal activity and blood flow. When there is a surge in neuronal activity, it triggers an increase in blood flow to the corresponding area, which is referred to as neurovascular coupling (NVC) [48]. NVC response has been demonstrated in the ONH blood flow and neuronal activity of RGCs with experiments investigating hemodynamic responses to flicker-light stimulation [49,50]. The NVC response in individuals with glaucoma is impaired [51], as the vasodilation response of the retina induced by flickering light [52] and the vasoconstrictive response to hyperoxia are attenuated [53]. Ischemia/hypoxia/perfusion instability may influence the astrocytes in the ONH and/or mitochondria of RGC axons, resulting in neurotoxic effects on RGCs [54,55,56].

### 3.4. Systematic Hemodynamics and Its Role in Glaucomatous Neurodegeneration

The role of systemic hemodynamics in glaucoma pathogenesis has been widely investigated. A “U-shaped” effect of BP on retinal function and structure has been revealed [8,57]. On the one hand, clinical studies have demonstrated that individuals with hypertension (HTN) [8,9] have a higher risk of developing glaucoma, with neural damage being more severe in glaucoma patients with HTN than those with normal BP [58]. Meanwhile, the treatment of HTN with beta-blockers can decrease the risk of glaucoma [59]. On the other hand, systemic hypotension and nocturnal dipping have also been associated with an increased risk of glaucoma [60]. The association between BP and neural deficits was shown to be mediated by decreased blood flow, increased vascular resistance, or lack of autoregulatory reserve [10,57,61]. Therefore, BP management in patients with concurrent systemic HTN and glaucoma is important. It is imperative to avoid excessive nocturnal BP dipping and to monitor potential retinal neuronal damage due to nocturnal hypotension induced by these treatments [11,60]. The recent TIME trial showed that the time of administration (morning or evening) for antihypertensive medication does not affect cardiovascular outcomes [62]. This suggests more flexibility in tailoring treatments for patients with concomitant glaucoma and HTN.

**Figure 2:**
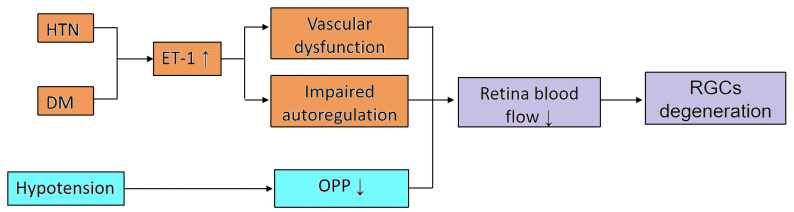
The proposed mechanism of systematic hemodynamic diseases in reducing retinal blood flow (DM: diabetes mellitus; HTN: systematic hypertension; ET-1: endothelin-1; OPP: ocular perfusion pressure; RGCs: retinal ganglion cells).

Apart from BP, it also has been reported that individuals with DM have a higher incidence of developing glaucoma [63]. Additionally, animal models have demonstrated that DM can trigger the degradation of RGCs [64,65]. One proposed mechanism is that DM [66,67] leads to a reduction in retinal blood flow, subsequently inducing retinal ischemia and hypoxia, and therefore RGC degeneration. In addition to exerting an influence upon systemic hemodynamics, DM can precipitate the onset of glaucoma through the elevation of IOP, a topic that is further discussed in Section 3.5. We summarize the systematic hemodynamic diseases contributing to reduced retinal blood flow and ganglion cell degeneration in Figure 2.

### 3.5. The Role of Vascular Abnormalities in Regulating IOP

IOP is the dynamic equilibrium between the production and drainage of AH [14,68]. The ciliary body in the posterior chamber secretes AH [69], which is primarily drained through the trabecular meshwork (TM) and collector canals before exiting into the episcleral veins and cavernous sinus [69]. Elevated IOP represents a significant etiological factor contributing to the onset and progression of glaucoma, while vascular abnormalities also precipitate elevated IOP, thereby fostering the pathogenesis of glaucoma [4,70].

It has been demonstrated that different vascular diseases are related to increased IOP. For instance, the pericyte loss of retinal blood vessels is a recognized marker of diabetic retinopathy (DR), which provokes capillary occlusion and overexpressed VEGF [70], potentially leading to neovascular glaucoma (NVG) [4,71,72]. In high-glucose conditions, the synthesis of extracellular matrix components including fibronectin is significantly increased, resulting in increased AH outflow resistance in TM, thus elevating IOP [73]. In addition, DM is considered to increase optic nerve head susceptibility to additional stress such as elevated IOP [7,74,75]. Arterial hypertension is another vascular disease that can increase IOP by elevating BP in the ciliary capillaries, leading to increased secretion of AH [76,77]. High BP can also raise episcleral venous pressure (EVP), which can increase resistance to AH drainage and further contribute to increased IOP [78,79]. Another vascular abnormality that can lead to increased IOP is hyperlipidemia. Studies have shown that hyperlipidemic patients are at an increased risk of glaucoma, potentially due to the increased viscosity of blood lipid levels, which in turn increases EVP [80,81]. Evidence supporting this hypothesis includes a lower incidence of glaucoma among patients receiving anti-hyperlipidemia therapy [82,83], as well as an increase in AH outflow facility observed in cultured porcine eyes treated with statins [84].

Sturge–Weber syndrome (SWS) is a rare, non-inherited neurovascular disorder characterized by abnormal vasculature in the brain, skin, and eye [85]. Glaucoma in SWS is another indication of elevated IOP caused by vascular abnormalities [86]. In glaucomatous eyes due to SWS, higher EVP was observed and thought to be the underlying pathophysiological mechanism of glaucoma [87,88]. Gonioscopy examination revealed reflux of blood in the Schlemm’s canal and episcleral hemangiomas, suggesting that Schlemm’s canal may be a component of hemangioma. Arteriovenous shunts in episcleral hemangioma may be the cause of elevated EVP, which consequently elevates IOP [88].

**Figure 3:**
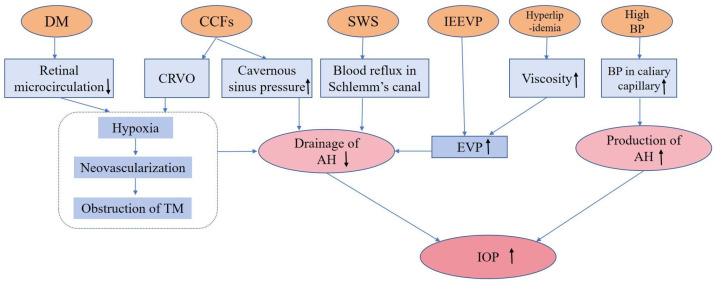
The possible roles of different vascular abnormalities in increasing IOP (DM: diabetes mellitus; CCFs: carotid-cavernous fistulas; SWS: Sturge–Weber syndrome; IEEVP: idiopathic elevated episcleral venous pressure; BP: blood pressure; TM: trabecular meshwork; AH: aqueous humor; EVP: episcleral venous pressure; IOP: intraocular pressure).

Carotid–cavernous fistulas (CCFs) are acquired vascular abnormalities with flow between the cavernous sinus and branches of either the external or internal carotid artery [89]. Elevated IOP and secondary glaucoma are common in CCFs [90]. Increased IOP in CCFs can be attributed to abnormal vascular shunts allowing carotid blood flow into cavernous fistulas, causing elevated pressure in the cavernous sinus and hindering the venous blood outflow from retinal and choroidal circulation, leading to anterior chamber (AC) shallowing and increased IOP [90,91,92]. Additionally, CCFs can result in neovascular glaucoma due to retinal hypoperfusion and ischemia [90,93,94]. While some cases of elevated EVP are due to identifiable etiologies, others are characterized as idiopathic elevated episcleral venous pressure (IEEVP) presenting with engorged episcleral veins, raised IOP, and/or glaucomatous visual field changes [94,95,96,97]. The elevated IOP due to vascular abnormities can cause compression of RGCs and their axons, as well as reduced ocular blood supply, leading to the development of glaucoma [15]. We summarize the different vascular abnormalities resulting in increased IOP in Figure 3.

### 3.6. Biomarkers Associated with Ocular Blood Flow in Glaucoma

ET-1, a key regulator of vascular autoregulation [98,99], has been found to be overexpressed in the retina of diabetic individuals [98,100], and increased expression of ET-1 has also been observed in the plasma of patients with high BP [101,102]. Overexpression of ET-1 may therefore play a role in the impaired autoregulation of retinal vessels. Furthermore, increased ET-1 expression is a biomarker that has been implicated in retinal vascular dysfunction, which is another potential mechanism contributing to reduced retinal blood flow [103,104]. This hypothesis is supported by the finding that an ET-1 blocker can increase retinal blood flow [105]. Clinical studies have reported that glaucoma patients have higher levels of plasma ET-1 compared to healthy controls [106], and those with deteriorating visual fields have higher plasma ET-1 levels than those with normal visual fields [107]. Animal experiments have also shown that chronic administration of low doses of ET-1 in primates and rabbits can cause glaucomatous changes [108,109]. Thus, ET-1 may serve as a useful biomarker for predicting glaucoma progression or as a target for pharmacological intervention.

An imbalance between the vasoconstrictor ET-1 and the vasodilator nitric oxide has been observed in glaucoma, leading to endothelial dysfunction and decreased ocular blood flow [30]. Nitric oxide is a molecule that dilates blood vessels and is thought to be involved in the regulation of ocular blood flow in glaucoma [110]. Several studies have shown that the availability of nitric oxide is reduced in glaucoma [110,111], leading to a shift in the balance between vasoconstriction and vasodilation and resulting in decreased blood flow in the optic nerve head [111]. Collectively, ET-1 and nitric oxide could be reliable biomarkers in predicting glaucoma progression and/or targeting pharmacological intervention from a vascular perspective.

### 3.7. Biomarkers Associated with Aqueous Humor Outflow or Trabecular Meshwork Function

VEGF is a primary factor contributing to vascular permeability and plays a pivotal role in the process of neovascularization [112]. In the context of NVG, an excessive quantity of VEGF is observed entering the AC through the posterior pole, leading to the development of neovascularization and fibrovascular membranes over the TM. This process results in the obstruction of the AH outflow through the TM, leading to elevated IOP, which can potentially lead to glaucomatous optic neuropathy [112].

Nitric oxide is generated by neuronal nitric oxide synthase and endothelial nitric oxide synthase in a Ca^2+^/calmodulin-dependent manner, which is essential for maintaining IOP homeostasis [113]. Neuronal nitric oxide synthase is predominantly expressed in ciliary processes and nerve endings, with high expression in the anterior segment of the eye, such as ciliary muscle, trabecular meshwork, Schlemm’s canal, and collecting channels [113,114]. These structures are crucial in regulating AH dynamics and IOP homeostasis in both physiological and pathological conditions [113]. Ocular hypertensive patients have reduced nitric oxide formation compared to healthy individuals, and exogenous nitric oxide administration has been shown to lower IOP in these patients [114]. Polymorphisms in endothelial nitric oxide synthase are associated with a higher risk of glaucoma [115]. Studies with endothelial nitric-oxide-synthase-deficient mice [116] or animals with impaired ganglion cell activity [117,118] show that these animals have higher IOP than their wild-type littermates, further demonstrating the importance of nitric oxide signaling in regulating IOP.

MMPs are enzymes that play a role in the breakdown of extracellular matrix proteins, and are thought to contribute to the IOP change in glaucoma [119]. The TM generates the main AH outflow resistance and its extracellular matrix is constantly remodeled by MMPs [120]. The activity of MMPs has been found to be positively correlated with the AH outflow rate. Specifically, increasing MMP activity has been shown to elevate the outflow rate, while inhibiting MMP activity leads to a decrease in the outflow rate [121]. A recent study conducted using a porcine model has reported similar results, demonstrating that reduced activity of MMP-2 and -9 is associated with elevated IOP [122].

Together, the biomolecules VEGF, nitric oxide, and MMPs hold promise as potential biomarkers for predicting the progression of glaucoma and as targets for pharmacologic intervention.

## 4. Glaucoma Can Also Be the Instigator of Retinal Vascular Abnormalities

Retinal vascular dysfunction has been observed as a secondary manifestation both in individuals diagnosed with glaucoma and animal models employed to study the disease, suggesting that glaucoma may also serve as the instigator of retinal vascular abnormalities [16]. These secondary manifestations encompass various alterations, such as a notable reduction in retinal vascular diameter [17], decreased retinal vascular density [18], disturbances in retinal vascular molecules [19], impaired autoregulation of blood flow, dysfunction of retinal arterioles [16], and instances of retinal vein occlusion [17,20]. In this section, we aim to provide a comprehensive and in-depth discussion of the aforementioned alterations observed in both glaucoma patients and animal models used for studying the disease.

### 4.1. Reduced Retinal Vascular Diameter in Glaucoma

A study conducted on individuals with early normal-tension glaucoma demonstrated that there were no significant differences in retinal arterial diameters compared to normal controls. This finding suggests that the narrowing of retinal arterioles observed in glaucoma patients is a consequence of the disease rather than being its underlying cause [123]. Further research conducted on adult Sprague Dawley rats involved the experimental elevation of IOP by cauterizing three episcleral veins. The findings from this study demonstrated a significant reduction in both vessel caliber and area within the glaucomatous optic nerves of the experimental rats [17]. In another glaucoma model, utilizing elevated IOP in Brown Norway rats through a vascular loop, an acute elevation of IOP caused a decrease in total retinal blood flow and average retinal blood vessel size [124]. One proposed mechanism underlying glaucomatous conditions suggests that the impaired RGCs necessitate a reduced blood supply, subsequently triggering retinal arteriolar constriction through the process of autoregulation [120,125,126,127,128]. This mechanism appears plausible, since retinal vascular constriction is observed not only in glaucoma but also in other optic nerve damage diseases [127,129,130,131,132].

### 4.2. Reduced Retinal Vascular Density in Glaucoma

In a case-control study, it was discovered that individuals afflicted with unilateral acute primary angle closure glaucoma (PACG) demonstrated a significant reduction in peripapillary retinal vessel density within affected eyes, as compared to contralateral unaffected eyes [18]. The notable decrease in vessel density was found to be significantly associated with visual field loss, thinning of the retinal nerve fiber layer, and ganglion cell complex in eyes affected by PACG. This association suggests that the loss of retinal vessel density is closely related to the presence and progression of glaucoma [18]. A series of case reports revealed that the reduction in IOP can lead to a re-increase in peripapillary capillary density in the retinal nerve fiber layer in ocular hypertensive and glaucomatous eyes [18]. Likewise, a prospective study demonstrated that trabeculectomy led to an improvement in peripapillary capillaries’ microvasculature observed through optical coherence tomography angiography in POAG patients [133]. However, this improvement proved incomplete as other studies showed that peripapillary retinal vessel density in resolved acute PACG eyes was still lower than the unaffected fellow eyes [18,134]. In a study conducted on C57BL/6J mice, transient elevated IOP was induced by injecting sodium hyaluronate into the AC. This manipulation resulted in a significant reduction in the number of capillary branches observed in both the superficial and intermediate vascular plexus of the retina [135]. The reduction in retinal capillaries observed in glaucomatous rats follows a similar pattern, with a more pronounced impact on capillary density in the inner layers of the retina in response to elevated IOP [136]. This phenomenon may provide an explanation for the observed decline in RGCs and thinning of the retinal nerve fiber layer during the progression of glaucoma.

### 4.3. Altered Retinal Vascular Molecules in the Retinas of Murine Models of Glaucoma

In experiments conducted on cultivated mouse retinas subjected to elevated intrachamber pressure, it was observed that the expression of tight adherens junction proteins, such as zonula occludens 1 (ZO-1), occludin, and the adherens junction protein VE-cadherin in retinal vessels, underwent alterations [19]. These findings strongly indicate that the integrity of the blood–retina barrier was compromised under conditions of elevated pressure [19]. In vivo and in vitro studies have demonstrated the expression of ß-III-tubulin, a neuron-specific biomarker, in both pericytes and endothelial cells in response to elevated IOP and intrachamber pressure, respectively [137]. This regulation of neuron-specific ß-III-tubulin is believed to be associated with retinal vascular remodeling under elevated IOP. Notably, the expression of ß-III-tubulin has also been observed during the remodeling of mesenteric vessels in rats, further supporting its involvement in vascular remodeling processes [138]. Furthermore, ß-III-tubulin is involved in oxidative stress [139], which can induce vascular endothelial dysfunction and impair the autoregulation of retinal arterioles [16].

### 4.4. Impaired Autoregulation and Arteriolar Dysfunction in the Retina of a Murine Model of Glaucoma

In a mouse model of glaucoma induced by unilateral cauterization of three episcleral veins [16,140], elevated IOP was observed to lead to impaired autoregulation and vascular dysfunction of retinal arterioles. This means that the ability of the retinal arterioles to regulate blood flow and maintain proper vascular function was compromised. [16]. The exact underlying mechanism for this phenomenon is believed to involve heightened oxidative stress and inflammation within the retina [16].

### 4.5. Glaucoma Is a Risk Factor for Retinal Vascular Occlusions

**Figure 4:**
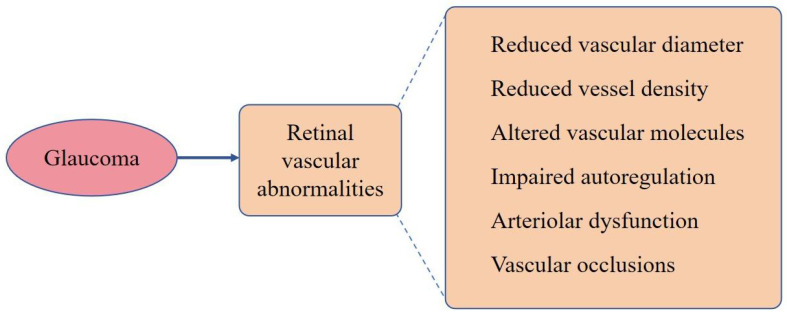
Different vascular abnormalities resulting from glaucoma.

Multiple studies have reported a notable association between glaucoma, specifically open-angle glaucoma, and an increased incidence of retinal vascular occlusions [141,142]. In a comprehensive Korean nationwide population-based retrospective study, it was observed that individuals diagnosed with open-angle glaucoma exhibited a significantly elevated incidence of retinal vascular occlusions when compared to the general population [20]. A retrospective case-control study conducted by Schwaber et al. (2018) [143] found that glaucoma was a risk factor for incident retinal vascular occlusions with an odds ratio of 6.19 (*p* < 0.001). However, some studies did not find this association [144,145]. A meta-analysis by Yin et al. (2019) [146] reported that glaucoma is a risk factor for retinal vascular occlusions with an odds ratio of 4.01. Subgroup analysis within the study indicated that glaucoma was significantly associated with various types of retinal vascular occlusions, including central retinal vein occlusion, branch retinal vein occlusion, and hemiretinal vein occlusion. Furthermore, the analysis of glaucoma subgroups revealed an association between open-angle glaucoma and central retinal vein occlusion, as well as between PACG and central retinal vein occlusion. However, there was no significant association observed between PACG and branch retinal vein occlusion. We have summarized the different vascular abnormalities resulting from glaucoma in Figure 4.

## 5. Conclusions

The introduction of the vascular theory, as well as the hemodynamics surrounding it, has spurred a growing body of scientific investigations aimed at unraveling the intricate relationship between vascular abnormalities and the underlying pathophysiology of glaucoma. It has become increasingly apparent that vascular abnormalities are closely associated with the observed neurodegeneration in glaucoma. However, it is important to recognize that glaucoma itself can initiate retinal vascular abnormalities. Nevertheless, given the multifactorial nature of glaucoma, it is acknowledged that vascular abnormalities represent just one of many contributing factors to the neurodegenerative processes observed in this disease. In addition to determining the causality between vascular abnormalities and glaucoma, longitudinal studies are necessary to disentangle the temporal association between loss of neural structure and loss of vascularization.

## Data Availability

Not applicable.

## Abbreviations

The following abbreviations are used in this manuscript:

AH	Aqueous humor
AC	Anterior chamber
BP	Blood pressure
CCFs	Carotid–cavernous fistulas
CRA	Central retinal artery
DM	Diabetes mellitus
DR	Diabetic retinopathy
ET-1	Endothelin-1
EVP	Episcleral venous pressure
HTN	Hypertension
IEEVP	Idiopathic elevated episcleral venous pressure
IOP	Intraocular pressure
MMPs	Matrix metalloproteinases
NVC	Neurovascular coupling
NVG	Neovascular glaucoma
OCTA	Optical coherence tomography angiography
ONH	Optic nerve head
OPP	Ocular perfusion pressure
PACG	Primary angle closure glaucoma
POAG	Primary open-angle glaucoma
RGCs	Retinal ganglion cells
SPCA	Short posterior ciliary artery
SWS	Sturge–Weber syndrome
TM	Trabecular meshwork
VEGF	Vascular endothelial growth factor
ZO-1	Zonula occludens 1
